# Association between junk food consumption and mental health problems in adults: a systematic review and meta-analysis

**DOI:** 10.1186/s12888-024-05889-8

**Published:** 2024-06-12

**Authors:** Hanieh-Sadat Ejtahed, Parham Mardi, Bahram Hejrani, Fatemeh Sadat Mahdavi, Behnaz Ghoreshi, Kimia Gohari, Motahar Heidari-Beni, Mostafa Qorbani

**Affiliations:** 1 Obesity and Eating Habits Research Center, Endocrinology and Metabolism Clinical Sciences Institute, Tehran University of Medical Sciences, Tehran, Iran; 2 Endocrinology and Metabolism Research Center, Endocrinology and Metabolism Clinical Sciences Institute, Tehran University of Medical Sciences, Tehran, Iran; 3 Social Determinants of Health Research Center, Alborz University of Medical Sciences, Karaj, Iran; 4 School of Medicine, Tehran University of Medical Sciences, Tehran, Iran; 5 Student Research Committee, Alborz University of Medical Sciences, Karaj, Iran; 6 Clinical Research Development Unit, Shahid Rajaei Educational & Medical Center, Alborz University of Medical Sciences, Karaj, Iran; 7 Non-communicable Diseases Research Center, Alborz University of Medical Sciences, Karaj, Iran; 8 Department of Biostatistics, Faculty of Medicine Sciences, Tarbiat Modares University, Tehran, Iran; 9 Department of Nutrition, Child Growth and Development Research Center, Research Institute for Primordial Prevention of Non-Communicable Disease, Isfahan University of Medical Sciences, Isfahan, Iran; 10 Chronic Diseases Research Center, Endocrinology and Metabolism Population Sciences Institute, Tehran University of Medical Sciences, Tehran, Iran

**Keywords:** Junk food, Mental health, Stress, Depression

## Abstract

**Background:**

Anxiety and depression can seriously undermine mental health and quality of life globally. The consumption of junk foods, including ultra-processed foods, fast foods, unhealthy snacks, and sugar-sweetened beverages, has been linked to mental health. The aim of this study is to use the published literature to evaluate how junk food consumption may be associated with mental health disorders in adults.

**Methods:**

A systematic search was conducted up to July 2023 across international databases including PubMed/Medline, ISI Web of Science, Scopus, Cochrane, Google Scholar, and EMBASE. Data extraction and quality assessment were performed by two independent reviewers. Heterogeneity across studies was assessed using the I^2^ statistic and chi-square-based Q-test. A random/fixed effect meta-analysis was conducted to pool odds ratios (ORs) and hazard ratios (HRs).

**Results:**

Of the 1745 retrieved articles, 17 studies with 159,885 participants were suitable for inclusion in the systematic review and meta-analysis (seven longitudinal, nine cross-sectional and one case-control studies). Quantitative synthesis based on cross-sectional studies showed that junk food consumption increases the odds of having stress and depression (OR = 1.15, 95% CI: 1.06 to 1.23). Moreover, pooling results of cohort studies showed that junk food consumption is associated with a 16% increment in the odds of developing mental health problems (OR = 1.16, 95% CI: 1.07 to 1.24).

**Conclusion:**

Meta-analysis revealed that consumption of junk foods was associated with an increased hazard of developing depression. Increased consumption of junk food has heightened the odds of depression and psychological stress being experienced in adult populations.

## Background

Psychological conditions such as bipolar affective disorder, eating disorders, anxiety disorders, and depressive disorders impose a considerable burden across the international community, adversely affecting quality of life [1, 2]. Psychological problems including depression, stress, and anxiety, also arise in association with some non-communicable diseases including cardiovascular disease (CVD), stroke, and cancer [3]. All of these mental health problems have adverse effects on health status, quality of life, and ability to work [4].

Genetics, socioeconomic status, exercise habits, diet, and nutritional status, are understood to be key contributors to the development of emotional or behavioral problems [5]. Food-mood relationships underpin well-known pathways, suggesting that unhealthy eating habits and poor nutritional status are correlated with various mental health problems and behavioral disturbances in adults [6]. This infers that mood and psychological health may be influenced by nutritional habits [7].

The world-wide consumption of junk foods, which include ultra-processed foods, fast foods, unhealthy snacks, and sugar-sweetened beverages, is increasing. The hallmarks of junk foods are that they have high levels of energy, fat, sugar, and salt, accompanied by low levels of micronutrients, fiber, and other bioactive compounds [8]. The low nutritional value of junk foods can alter inflammatory pathways, leading to an increase in biomarkers for oxidative stress and inflammation, which contribute to biological changes associated with mental health disorders. In vitro studies have demonstrated that junk food consumption can negatively affect the brain and mental health [9, 10].

However, the findings of epidemiological studies are inconsistent. Some studies showed the significant association between junk foods consumption and mental health disorders. However, other studies did not mention any relationship [4, 11, 12]. The aim of this study is to examine the relationship between junk food consumption and mental health disorders in adults by conducting a systematic review and meta-analysis of published studies to date.

## Methods

The current systematic review and meta-analysis study was conducted according to the PRISMA 2020 statement (Preferred Reporting Items for Systematic Reviews and Meta-Analyses) [13, 14], included studies assessing the relationship between junk food consumption and mental health in adults.

### Search strategy

A systematic literature search was conducted in PubMed/Medline, ISI Web of Science, Scopus, Cochrane, Google Scholar, and EMBASE up to July 2023. The following keywords were used in this search: “sweetened drink*” OR “sweetened beverage*” OR snack* OR “processed food*” OR “junk food*” OR “soft drinks” OR “sugared beverages” OR “fried foods” OR “instant foods” OR sweets for junk food consumption and “mental health” OR depression OR stress OR anxiety OR “sleep dissatisfaction” OR “sleep disorders” OR happiness OR wellbeing for mental health status. In PubMed, keywords were searched through [tiab] and [MeSH] tags. Articles were required to be written in English language; there was no limitation regarding the year of publication. The reference lists of included papers were also examined to avoid missing other published data.

### Inclusion and exclusion criteria

Two investigators independently screened the articles retrieved during the literature search. Publications that fulfilled the following criteria were eligible for inclusion: (1) observational studies that were conducted in adults (cohort, case-control, cross-sectional); and (2) studies that examined the relationship between junk food consumption and mental health status. We excluded letters, comments, reviews, meta-analyses, ecological, in vitro, and pre-clinical studies, as well as duplicate studies.

### Data extraction

For each eligible study, the following information was extracted: first author, year of publication, study design, country, age range, gender, sample size, type of junk food, dietary assessment tool, mental health parameters, mental health assessment tool, study quality score, effect sizes and measures, and covariates.

It should be noted that in the present study, junk food intake was considered using four categories: (i) sweet drinks (fruit-flavored drinks, sweetened coffee, fruit juice drinks, sugared coffee and tea, energy drinks, cola drinks, beverages, soft drinks, lemonade, and soda), (ii) sweet snacks (total sugars, added sugars, sweetened desserts, fatty/sweet products, ice cream, chocolate, artificial sweeteners, sweet snacks, dessert, sauces and dressings, candy, patterns of consumption of sweet, high fat and sugary foods, biscuits and pastries, cakes, pie/cookies, and baked goods), (iii) snacks (including snacks, sauces/added fats, fast food, fast-food pattern, western diet pattern, snacking and convenience pattern, fried foods, fried potato, crisps, salty snacks, convenience pattern, instant foods), and (iv) total junk foods (all types of junk foods).

### Quality assessment of studies

The quality of the included studies was examined using the Newcastle-Ottawa Scale (NOS) [15, 16]. The NOS assigns a maximum of 9 points to each study: 4 for selection, 2 for comparability, and 3 for assessment of outcomes (for cohort study) or exposures (for case-control study).

The maximum score for cohort and case-control studies were 9 and for cross-sectional studies were 7. In the current analysis, the quality of studies is defined good if the studies get 3 or 4 stars in the selection domain AND 1 or 2 stars in the comparability domain AND 2 or 3 stars in the outcome/exposure domain. Besides, fair quality is defined as 2 stars in the selection domain AND 1 or 2 stars in the comparability domain AND 2 or 3 stars in the outcome/exposure domain and finally, poor quality is defined for 0 or 1 star in the selection domain OR 0 star in the comparability domain OR 0 or 1 star in the outcome/exposure domain.

All steps including searching, article screening, data extraction, and quality assessment of articles were independently performed by two investigators. Disagreements between the two investigators were resolved by discussion to reach consensus.

### Statistical analysis

The results of the current quantitative synthesis are presented as hazard ratios (HRs) or odds ratios (ORs) and 95% confidence intervals (95% CI). STATA version 14.0 (StataCorp, College Station, TX) software was used to perform the meta-analysis. We conducted meta-analysis whenever at least two studies investigated similar associations between junk food consumption and mental health problems.

I^2^ statistic and chi-square-based Q-test were used for the assessment of heterogeneity. In the current study, a lack of heterogeneity was inferred when the p-value of chi-square-based Q-test exceeded 0.10. Fixed models were used to pool HRs and ORs when the heterogeneity p-value was higher than 0.10. Random models were used to pool the ORs whenever the heterogeneity p-value was equal to or less than 0.10, followed by Galbraith analysis and sensitivity analysis. Subgroup analysis was also conducted to identify the source of heterogeneity. Publication bias was measured using Begg’s test or Egger’s test and considered substantial whenever the resulting p-value was < 0.1.

## Results

### Systematic search results

**Fig. 1:**
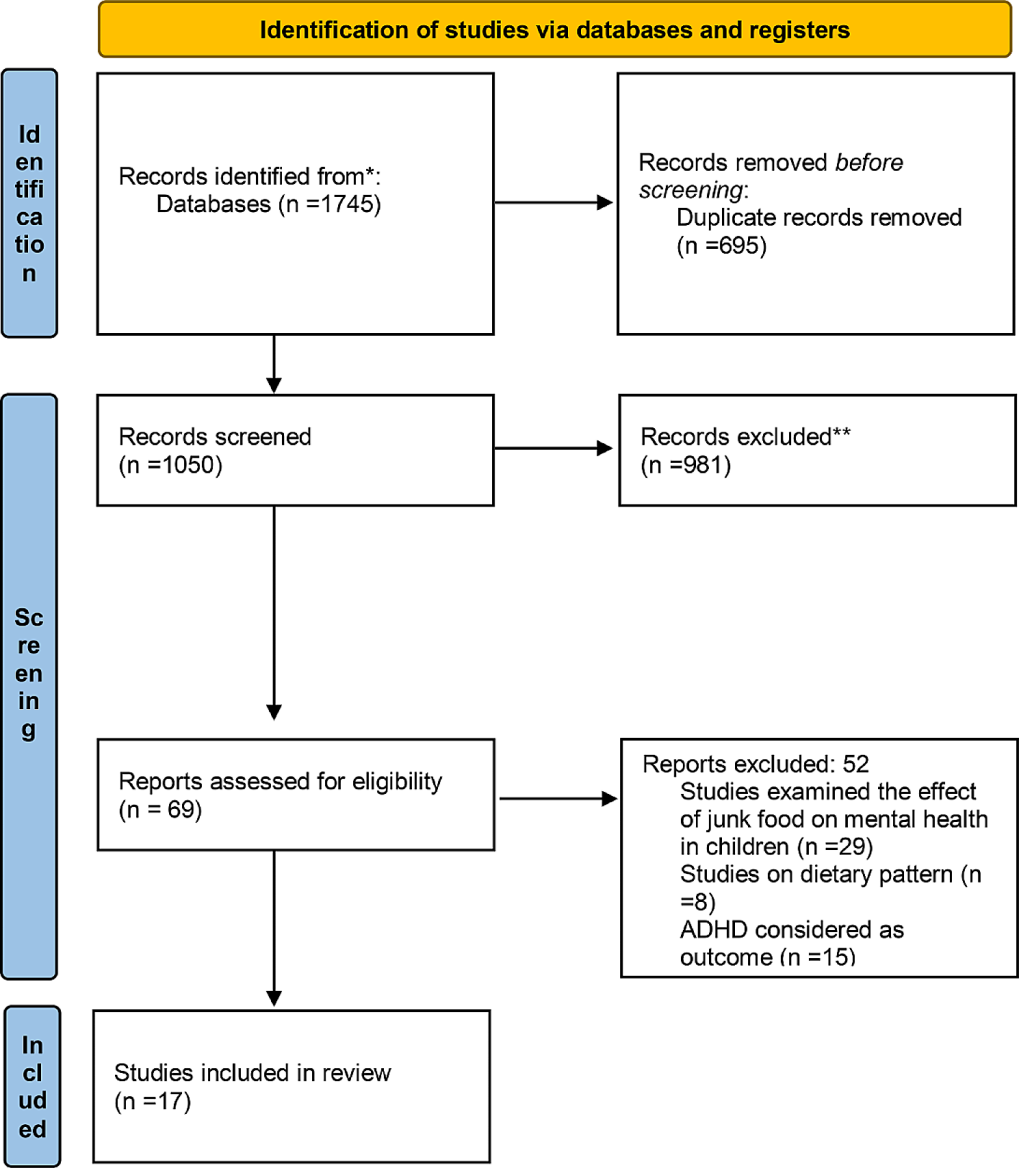
The PRISMA flowchart for the process of study selection

The flow diagram for the process of study selection is shown in the PRISMA flowchart (Fig. 1). Based on the initial search, we found 1745 papers. After removal of duplicate documents and title and abstract screening, 69 articles remained for more detailed assessment. Full texts of these papers were reviewed carefully by three researchers, with 17 articles satisfying the eligibility requirements for inclusion in the systematic review and meta-analysis.

### Characteristics of the included studies

Seventeen studies evaluating a total of 159,885 participants were included in our quantitative synthesis. A considerable number of participants were female, with seven articles restricted to female participants. Most of the included studies were cross-sectional (58.82%), with the remaining seven (47.05%) being cohort studies. It should be noted that Reinks et al. (2013) presented both cross-sectional and longitudinal data. Reinks et al. (2013) and ten other papers (64.70% in total) assessed depression as an outcome. Nine (52.94%) of studies assessed anxiety or stress as outcomes. In terms of dietary exposures, various types of junk foods such as ultra-processed food, beverages, and snacks were evaluated across the 17 studies. Table 1 illustrates detailed characteristics of records including the age of participants and provenance of studies. All of the included studies have good quality.

**Table 1:** Characteristics of the included studies

Number		Author/ (year)	Study characteristics					Psychological outcomes		Dietary exposures		QA
			Design	Provenance	Sample size	Age (range or mean)	Male Gender *n* (%)	psychological condition	Assessment tool	Type of junk food	Assessment tool (Practical definition)	
1		Adjibade, 2019	Cohort	France	26,730	47.26 ± 14.17	6350 (23.75)	Depression	CES-D (0–60) validated cut-offs (CES-D score ≥ 17 for men and ≥ 23 for women)	Ultra-processed food	Manufactured food products containing numerous ingredients as well as additives such as hydrogenated oils, non-sugar sweeteners, modified starch, flavoring agents, emulsifiers, humectants, colors, and other additives used for cosmetic purpose measured based on web-based dietary record platform validated for self-administration	7
										Beverages, fatty/sweet products, snacks, and sauces/ added fats	Percentage of dietary intake based on a web-based dietary record platform validated for self-administration	
2		Almajwal, 2016	cross-sectional	Saudi Arabia	395	NR	0 (0.0)	Stress	The perceived stress scale	Eating styles including, Restrained, Emotional, and External	The Dutch Eating Behavior Questionnaire self-reported questionnaire.	6
										Eating fast food	frequency of eating fast food per week	
3		Camiller, 2014	Cohort	France	30,240	46.2 ± 13.9	7378 (24.40)	Depressive symptoms	Validated French version of the Center for Epidemiologic Studies Depression Scale (CES-D)	Sugar-sweetened soft drinks	French version of the revised 21-item Three-Factor Eating Questionnaire	7
4		Canuto, 2021	cross-sectional	Brazil	539	33.6 ± 8.6	0 (0.0)	Perceived stress score	10-item Perceived Stress Scale (PSS-10)	Snack and fast-food	Validated qualitative food frequency questionnaire comprising 53 food items	8
5		Chaplin, 2011	Cross sectional	United Kingdom	870	45	75 (8.62)	Stress in life in general, work stress, cognitive failure outside work, minor injury outside work, and minor injury at work	Researcher-made validated questionnaire	Unhealthy snack	A factor analysis of snacking behavior, consisted of the sum of frequency of snacking of chocolate, crisps and biscuits, measured using a Likert scale	6
6		Coletro, 2022	Cross-sectional	Brazil	1693	NR	827 (48.9)	Anxiety symptoms	The Generalized Anxiety Disorder 7-item (GAD-7)	consumption of ultra-processed foods	Assessed using a qualitative food frequency questionnaire (FFQ), validated in Brazilian population referring to consumption in the last 3 months	7
								Depression symptoms	Patient Health Questionnaire-9 (PHQ-9)			
7		Crawford, 2011	Cross sectional	United States	626	45–54	0 (0.0)	Depression	The Center for Epidemiological Studies-Depression (CES-D) scale (scoring 16 or higher out of 20 items)	Fast food	Frequency of fast-food intake was measured by self-report	7
8		Gómez-Donoso, 2019	Cohort	Spain	14,907	36.7 ± 11.7	0 (0.0)	Depression	Clinical diagnosis or antidepressant medication use	Ultra-processed food	Frequency of intake of carbonated drinks, processed meat, biscuits (cookies), candy (confectionery), ‘instant’ packaged soups and noodles, sweet or savory packaged snacks, and sugared milk and fruit drinks	7
9		Le Port, 2012	cohort	France	12,404	M:45.0 ± 2.9 F: 42.2 ± 4.2	9272 (74.75)	Depression	The 20 items scale of The Center for Epidemiological Studies-Depression (CES-D)	Western diet, fat-sweet, snacking, and dessert	35-item qualitative Food Frequency Questionnaire (FFQ) for twenty food groups	8
10		Lim, 2020	longitudinal study	United States	912	28.7 ± 0.3	0 (0.0)	Chronic stress	10-item Perceived Stress Scale (PSS-10)	Excess fat and soda intake	Frequency of intake of instant noodle, frozen, canned or microwave foods, potato chips, corn chips and tortilla chips, McDonald’s, KFC, Pizza Hut/Bi Sheng Ke	7
11		Liu, 2007	Cross sectional	China	2541	20.4	1470 (57.85)	Stress	10-item Perceived Stress Scale (PSS-10)	Ready to eat food or snack	Not validated food frequency questionnaire regarding the previous month	6
								Depression score	The 20 items scale of The Center for Epidemiological Studies-Depression (CES-D)			
12		Nitturi, 2021	Cross sectional	United States	107	49.3 ± 11.6	22 (20.56)	Anxiety sensitivity	The Anxiety Sensitivity Index (ASI)	Unhealthy Supersized fast food	Researcher-made validated questionnaire	7
13		Rienks, 2013	Longitudinal and cross-sectional data driven from a prospective study	Australia	8369 for cross-sectional and 6060 for longitudinal analysis	50–55	0 (0.0)	Depression	The 10-items scale of Centre for Epidemiologic Studies Depression (scores ranges from 0–30, participants with a score of 10 or higher were considered depressed	Meat and processed meat and high fat and sugar pattern	validated food frequency questionnaire asking regarding 74 foods and six alcoholic beverages over the last 12 months	8
14		Sangsefidi, 2020	Cross-sectional (Data from The recruitment phase of a cohort)	Iran	9965	20–69	4921 (49.7)	Depression, Anxiety, and Stress	The Iranian validated version of depression, anxiety, and stress scale questionnaire 21 (DASS 21), a well-known short version of self-report	Sweetened drinks, Fast foods, Canned foods, Fried foods, and Snacks	Not validated Food Frequency Questionnaire (FFQ), asking about the last year (results were divided into three groups of never, once, or more than once per week)	7
15		Sousa, 2013	Cross sectional	Brazil	46,785	20–59	22,410 (47.9)	Depression	Patient Health Questionnaire-9 (PHQ-9)	Sugar sweetened beverage, Sweets, and Snacks	Not validated Food Frequency Questionnaire (FFQ), asking about the last week (High consumption was considered when a participant reported 5 times or more intake per week)	7
16		Xia, 2017	Case control	China	2702	Control:45.84 Case: 46.08	1450 (53.66)	depression	Chinese version of Zung Self-Rating Depression Scale (SDS)	Sugared beverages, Salted foods	Not validated food frequency questionnaire (FFQ), consisted of 81 items, including 7 frequency categories ranging from “almost never eat” to “twice or more per day”	7
17		Zenk, 2014	Prospective cohort	United States	100	44.3 ± 10.5	0 (0.0)	Stressful events, within-person stressful social interaction, and between-person stressful social interaction	Researcher-made not validated questionnaire	Snack food intake	Not validated web-based momentary surveys via study provided smartphones	6

F, Females; M, Males; NR, Not Reported

### Qualitative synthesis

Most of the included studies concordantly showed at least a single significant link between junk food consumption and psychological outcomes. This was despite their use of different measures of association, dissimilar exposure duration and outcomes, and heterogenous definitions, all of which made it challenging to draw conclusions from the qualitative synthesis (summarized in Table 2). Nevertheless, findings from some studies were discordant. For instance, while Sangsefidi et al. (2020) and Chaplin et al. (2011) demonstrated a significant association between stress and snack intake, Almajwal et al. (2016) and Zenk et al. (2014) reported non-significant findings, despite the use of similar measures of association and comparable adjustments for covariates. Although a notable number of studies showed a significant link between junk food intake and psychological disorders, the level of disagreement across studies meant that a meta-analysis was essential in order to clarify this relationship.

**Table 2:** Findings of qualitative synthesis

NO	Author/ (year)	Psychological outcomes	Dietary exposures		Measure of association	Study findings		Confounders
1	Adjibade, 2019	Depression	Ultra-processed food (Q4/Q1)		HR (95% CI)	1.29 (1.13–1.47)*		Age, sex, BMI, marital status, education, occupational categories, household income per consumption unit, residential area, number of 24-h dietary records, inclusion month, energy intake without alcohol, alcohol intake, smoking, PA, dietary patterns, intakes of lipids, sodium, and carbohydrates.
			Beverages (Q4/Q1)			1.25 (1.13–1.38)*		Age, sex, marital status, educational level, occupational categories, household income per consumption unit, residential area, energy intake without alcohol, number of 24-h dietary records, inclusion month, smoking status, physical activity, BMI, health events during follow-up (cancer, type 2 diabetes, hypertension and cardiovascular events) and quantity of the equivalent food group.
			Fatty/sweet products (Q4/Q1)			1.08 (0.96–1.22)		
			Snacks (Q4/Q1)			1.10 (0.98–1.24)		
			Sauces/ added fats (Q4/Q1)			1.23 (1.10–1.39)		
2	Almajwal, 2016	Stress	Eating styles	Restrained	Spearman’s correlation coefficients	0.115	p-value < 0.05	Age, gender, education, experience, and marital status
				Emotional		0.128		
				External		0.170		
		Stress (low vs. high)	Eating fast food (low)	Never or rarely	Number of participants (Chi-square)	42 (18.7)	χ2 = 14.99; *p* = 0.002	Not adjusted
				Sometimes		169 (75.1)		
				Often		9 (4.0)		
				Almost everyday		5 (2.2)		
			Eating fast food (high)	Never or rarely		29 (13.7)		
				Sometimes		145 (68.7)		
				Often		29 (13.7)		
				Almost everyday		8 (3.8)		
			Eating snacks (low)	Never or rarely		28 (12.4)	χ2 = 0.43; *p* = 0.934	
				Sometimes		147 (65.3)		
				Often		37 (16.4)		
				Almost everyday		13 (5.8)		
			Eating snacks (high)	Never or rarely		28 (13.3)		
				Sometimes		141 (66.8)		
				Often		32 (15.2)		
				Almost everyday		10 (4.7)		
3	Camilleri, 2014	Depressive symptoms	Sugar-sweetened soft drinks		OR (95% CI)	Male	1.02 (0.72, 1.44) *	Age, total daily energy intake, BMI, educational level, employment status, marital status, smoking status, physical activity, history of dieting, and season of completing the 24-h records
						Female	1.03 (0.83–1.27) *	
4	Canuto, 2021	Perceived stress score	snack and fast-food		PR (95% CI)	1.28 (1.04–1.56) *		Age, skin color, marital status, education, BMI, wake time and work shift
5	Chaplin, 2011	Stress in life in general	Unhealthy snack		OR (95% CI)	1.57 (1.15–2.16) *		Smoking, alcohol, sleep problems, age, sex, breakfast frequency, exposure to physical hazards and working hours score, Demand- control- support score, and Effort-Reward imbalance score.
		Work stress				1.61 (1.13–2.29) *		
		Cognitive failure outside work				1.51 (1.07–2.12)*		
		Minor injury outside work				1.54 (1.14–2.09)*		
		Minor injury at work				1.95 (1.40–2.71)*		
6	Coletro, 2022	Anxiety symptoms	consumption of ultra-processed foods		PR (95% CI)	1.5 (1.03–2.3) *		Sex, age, marital status, educational background, family income and medical diagnosis of depression or anxiety disorders
		Depression symptoms				1.5 (1.1–2.1) *		
7	Crawford, 2011	Depression (present/ absent)	Fast food		OR (95% CI)	F: 1.54 (1.06–2.25)*		Age, race, marital status, education, annual household income, BMI, smoking, leisure PA, alcohol use, ADD.
8	Gómez-Donoso, 2019	Depression (incidence)	Ultra-processed food (Q4/Q1)		HR (95% CI)	1.33 (1.07–1.64)*		Sex, age, year, baseline BMI, total energy intake, PA, smoking, marital status, living alone, employment status, working hours per week, health-related career, years of education, adherence to Trichopoulou’s MeDiet Score, baseline self-perception of competitiveness, anxiety, dependence levels.
9	Le Port, 2012	Depression	Western diet (Q4/Q1)		OR (95% CI)	M: 1.36 (1.19–1.54)*		Age, employment position at 35, professional activity, BMI, marital status, PA, tobacco, smoking, alcohol intake.
			Fat-sweet (Q4/Q1)			M: 1.49 (1.30–1.71)*		
			Snacking (Q4/Q1)			M: 1.50 (1.32–1.71)* F: 1.43 (1.16–1.76)*		
			Dessert (Q4/Q1)			F: 1.03 (0.84–1.26)		
10	Lim, 2020	Chronic stress (yes/ no)	Excess fat/ soda intake		PR (95% CI)	1.39 (1.05–1.84)*		Demographic characteristics, total dietary calorie intake.
11	Liu, 2007	Perceived stress score	Ready to eat food (low/ high frequency)		OR (95% CI)	0.69 (0.57–0.84)*		Sex, city, perceived weight, smoking.
			Snack food (low/ high frequency)			0.75 (0.59–0.94)*		
		Depression score	Snack food (low/ high frequency)			0.73 (0.58–0.93)*		-
			Ready to eat food (low/ high frequency)			0.70 (0.57–0.86)*		Sex, grade, city, perceived weight, smoking, alcohol use.
			Fast food (low/ high frequency)			0.40 (0.12–1.37)*		
12	Nitturi, 2021	Anxiety sensitivity	Unhealthy/Supersized fast food (always/ never)		OR (95% CI)	1.05 (1.01–1.08)*		Sex, age, and BMI
13	Rienks, 2013	Prevalence of depression	Meat and processed meat		OR (95% CI)	1.06 (0.99–1.13)		Energy, smoking, PA, ability to manage on available income, occupation status, education, marital status, mean stress score, BMI category.
			High fat and sugar pattern			1.02 (0.96–1.09)		
		Incidence of depression	Meat and processed meat			1.09 (0.98–1.21)		
			High fat and sugar pattern			1.08 (0.96–1.20)		
14	Sangsefidi, 2020	Depression	Sweetened drinks (never/ Once or more per week)		OR (95% CI)	0.76 (0.59–0.96)*		Age, education level, PA, history of chronic diseases, smoking and BMI
		Anxiety				0.76 (0.62–0.93)*		
		Stress				0.63 (0.48–0.82)*		
		Depression	Fast foods (never/ Once or more per week)			1.61 (1.18–2.203)*		
		Anxiety				1.19 (0.908–1.56)		
		Stress				1.28 (0.88–1.86)		
		Depression	Canned foods (never/ Once or more per week)			1.12 (0.78–1.61)		
		Anxiety				1.13 (0.83–1.54)		
		Stress				1.05 (0.69–1.59)		
		Depression	Fried foods (never/ Once or more per week)			1.03 (0.69–1.52)		
		Anxiety				1.01 (0.73–1.39)		
		Stress				2.47 (1.46–4.18)*		
		Depression	Snacks (never/ Once or more per week)			1.36 (1.01–1.84)*		
		Anxiety				1.99 (1.55–2.56)*		
		Stress				1.73 (1.23–2.45)*		
15	Sousa, 2013	Depression	Sugar sweetened beverage (regular/no)		OR (95% CI)	1.13 (0.99–1.29)		Age, sex, race/color, education, living with spouse, PA, alcohol consumption, tobacco use.
			Sweets (regular/no)			1.53 (1.33–1.76)*		
			Snacks (regular/no)			1.52 (1.21–1.90)*		
16	Xia, 2017	Depression	Sugared beverages		OR (95% CI)	1.09 (0.87, 1.35)		For other food groups intake
			Salted foods			1.13 (0.90, 1.41)		
17	Zenk, 2014	Stressful event	Snack food intake		OR (95% CI)	1.24 (0.97, 1.60)		Age, education (high school diploma, GED, or less; associate’s degree or some college; bachelor’s degree; graduate or professional degree), employment status (unemployed/other including retired or Disabled; employed part-time, employed full-time), annual per capita household income (approximate tertiles: <$7500, $7500-18,749, ≥$18,750), automobile ownership, and body mass index (BMI), calculated as interviewer-measured weight (kg/[height (m)]2)
		Within-person stressful social interaction				0.90 (0.67, 1.22)		
		Between-person stressful social interaction				1.10 (1.00, 1.22)		

*Abbreviations* SES: socioeconomic status, ST: screen time, NR: not reported, ADHD: Attention deficit hyperactivity disorder, T: tertile, Q: quantile, PA: Physical Activity, ADD: use of anti-depressive drugs, CF: cognitive function, OR: odds ratio, RR: relative risk, HR: hazard ration, SMD: standardized mean difference

### Quantitative synthesis

#### Pooling OR in cross-sectional studies

**Fig. 2:**
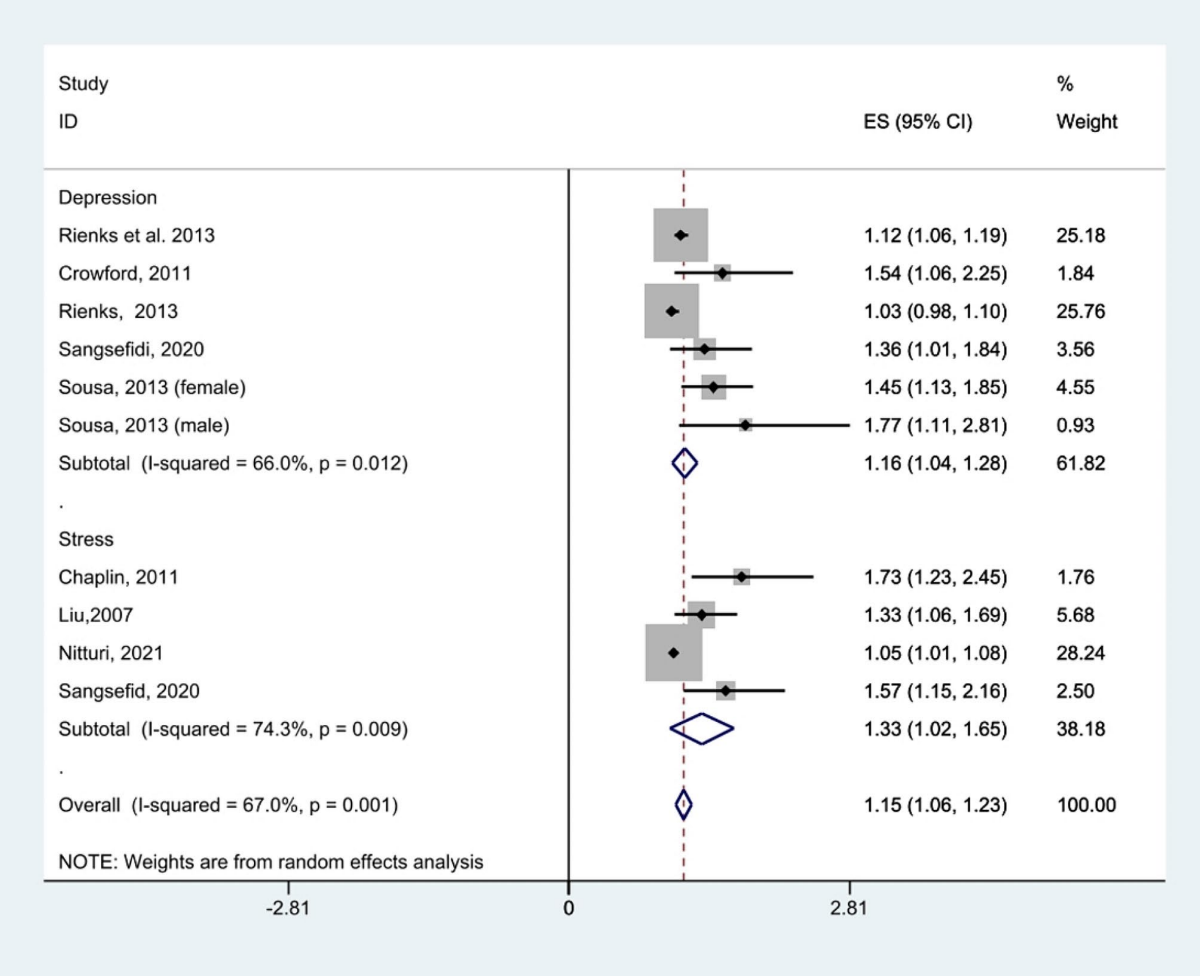
Junk food consumption (unhealthy snacks and sweetened beverages) and odds of having depression and stress in cross-sectional studies

**Fig. 3:**
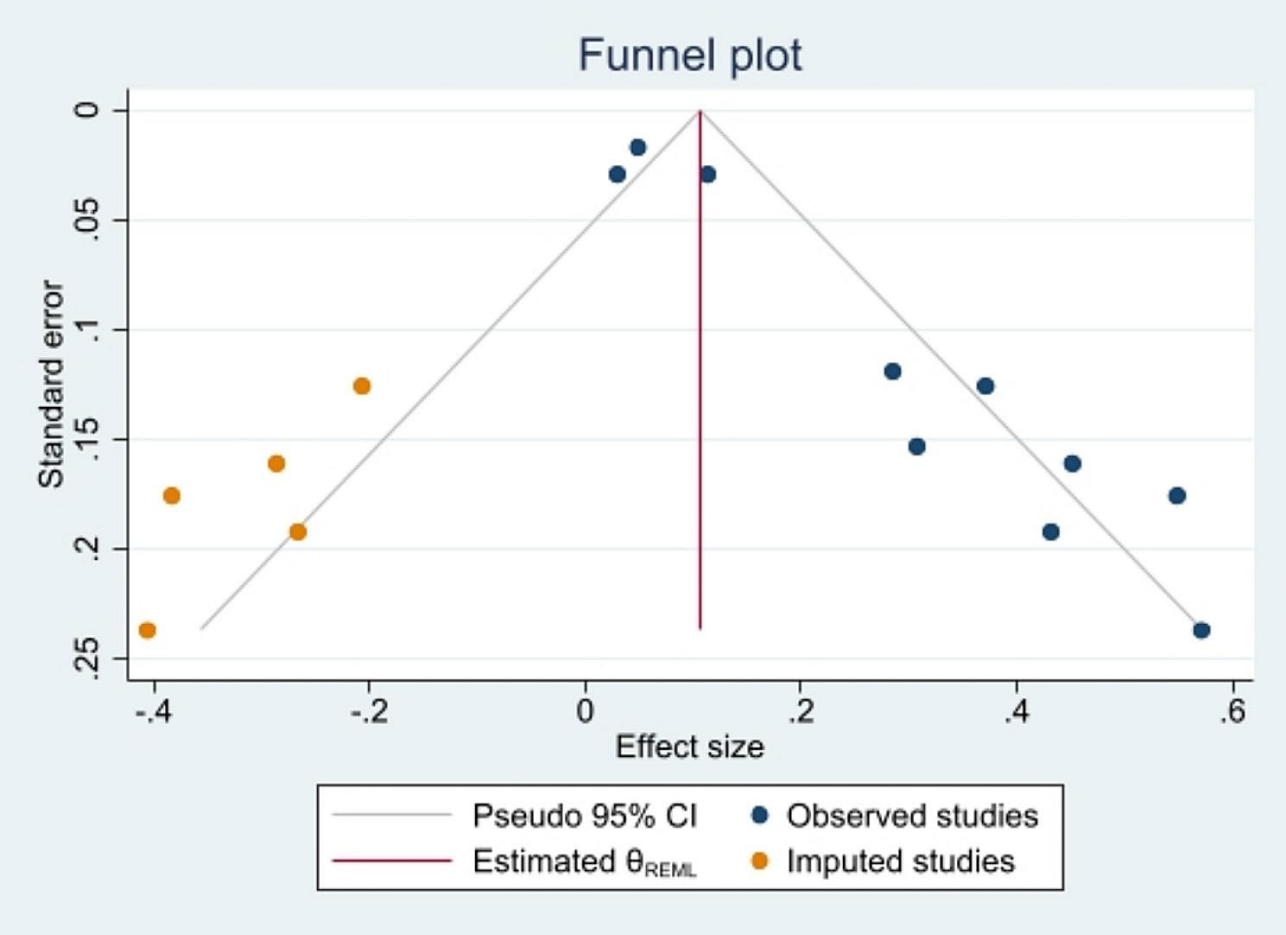
Funnel plot, using data from cross-sectional studies investigating the association between junk food consumption and mental health problems

Four cross-sectional studies (*n* = 13,500) demonstrated that junk food consumption was associated with increased stress (pooled OR = 1.33, 95% CI: 1.02 to 1.65). This finding shows a significant association; however, a notable level of heterogeneity was observed (I² = 74.3%, *p* = 0.009) (Fig. 2; Table 3). Also, six cross-sectional studies, including 74,127 participants, illustrated a significant association between junk food consumption and depression, with a pooled OR of 1.16 (95% CI: 1.04 to 1.28) (Fig. 2). Overall, junk food consumption indicated a significant association with increased odds of mental health problems (OR = 1.15, 95% CI: 1.06 to 1.23). The Egger’s test for small-study effects indicated evidence of publication bias (*p* > 0.001). To address this bias, a trim and fill analysis was conducted, resulting in an adjusted OR of 1.11 (95% CI: 0.95 to 1.30). Funnel plot is presented in Fig. 3.

**Table 3:** Findings of Quantitative Synthesis

No	Study Type	Measure of Association	Outcome	Number of Studies	Sample Size	Pooled Results Measure (95%CI)	Heterogeneity			
							Chi-Squared	I^2^	p-value	Model
1	Cross-sectional	OR	Stress	4	13,500	1.333 (1.018 to 1.649)	11.68	74.3%	0.009	Random Effects
2			Depression	6	74,127	1.161 (1.039 to 1.283)	14.72	66.0%	0.012	Random Effects
3			Mental Disorder (Overall)	10	87,627	1.148 (1.065 to 1.232)	27.26	67.0%	0.001	Random Effects
4			Mental Disorder (After trim and fill)	15	87,627	1.11 (0.95–1.30)	N/A	N/A	N/A	Random Effects
5		PR	Stress	2	2,232	1.312 (1.071–1.552)	0.39	0.0%	0.530	Fixed
6	Cohorts	OR	Depression	8	46,821	1.152 (1.062–1.241)	24.81	71.8%	0.001	Random
7			Mental Disorder (Overall)	9	46,921	1.156 (1.070–1.242)	25.66	68.8%	0.001	Random
8		HR	Depression	2	41,637	1.300 (1.154 to 1.446)	0.06	0.0%	0.813	Fixed

CI, Confidence Interval; OR, Odds Ratio; PR, Prevalence Ratio; HR, Hazard Ratio

### Pooling PR in cross-sectional studies

**Fig. 4:**
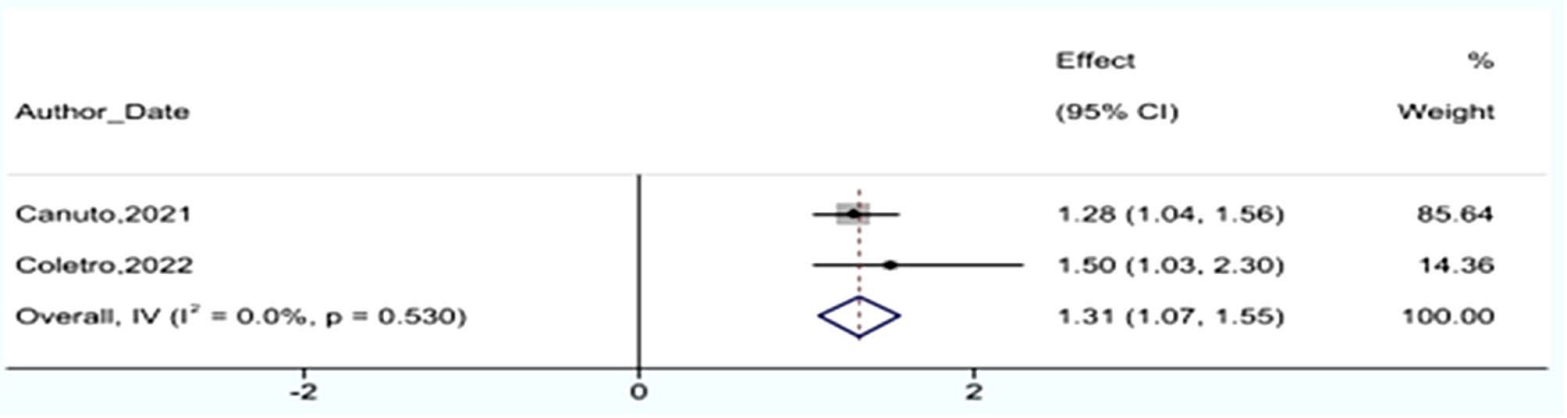
Association between junk foods consumption and having stress in cross-sectional studies

Two cross-sectional studies focusing on stress with a combined sample size of 2,232 participants reported a PR of 1.31 (95% CI: 1.07–1.55) (Fig. 4).

### Pooling OR in cohort studies

**Fig. 5:**
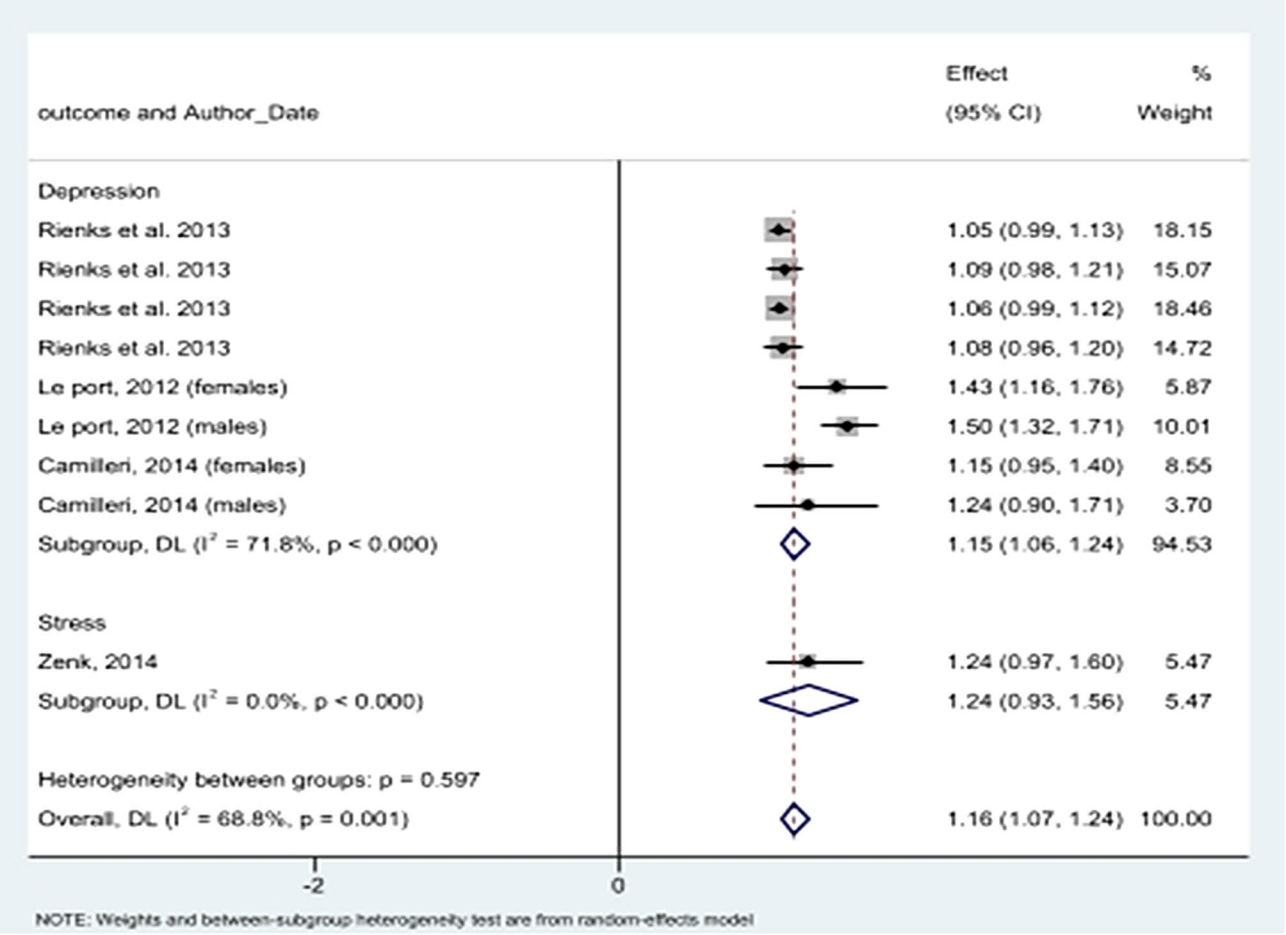
Association between junk foods consumption and having mental health problems in cohort studies

Pooling results of cohort studies showed that junk food consumption significantly increases the odds of depression by 15% (OR = 1.15; 95% CI: 1.06 to 1.24). After inclusion of the single cohort study that considered stress as its outcome, the overall OR of junk foods consumption and mental disorders was 1.16 (OR = 1.16, 95% CI: 1.07 to 1.24) (Fig. 5).

**Fig. 6:**
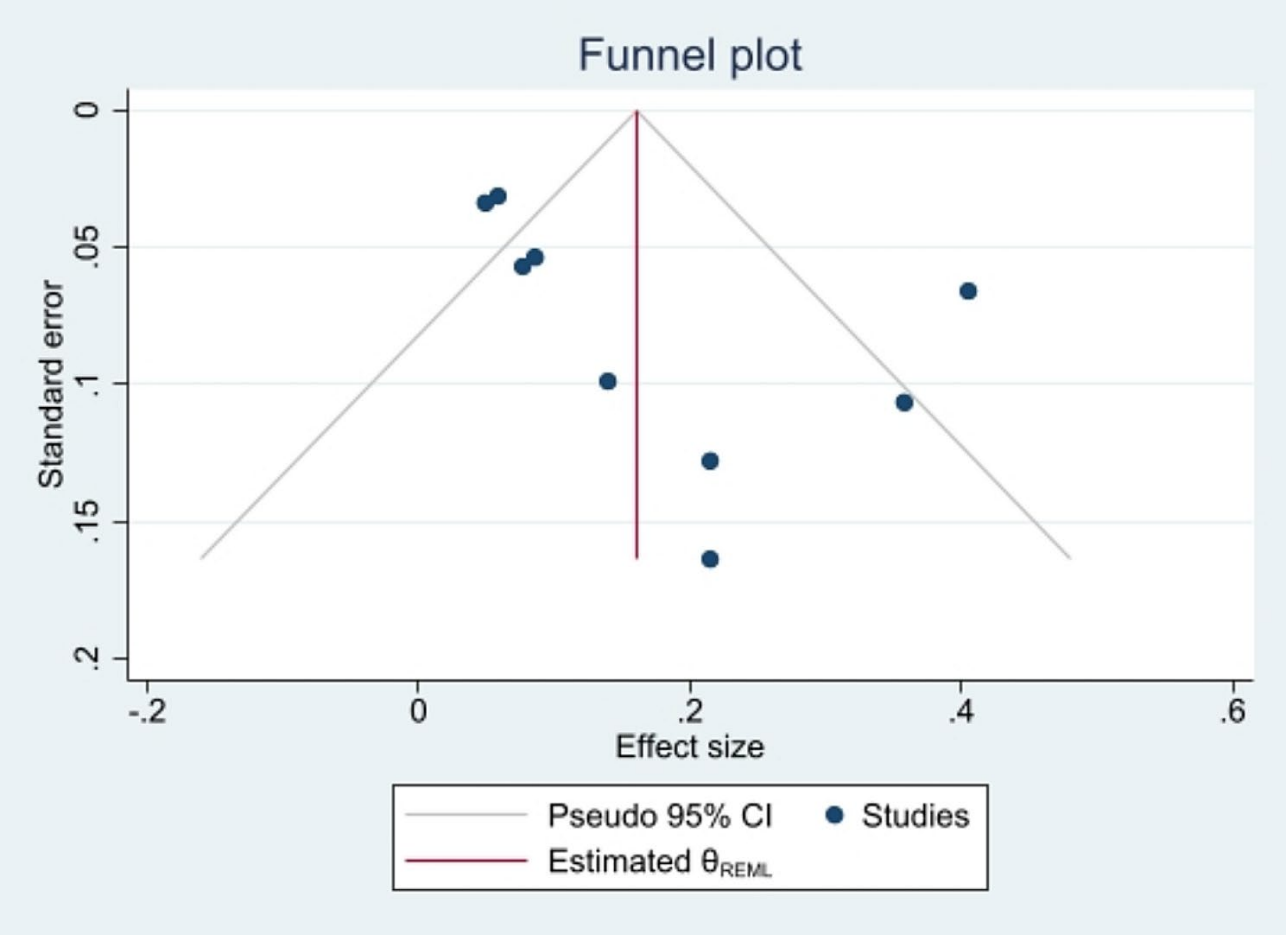
Funnel plot, using data from cohort studies investigating the association between junk food consumption and mental health problems

Although Egger’s test for small-study effects yielded a bias coefficient of 2.53, standard error of 1.19, and a p-value of 0.07, trim and fill analysis did not impute any studies, and the overall OR remained unchanged. Figure 6 demonstrates the funnel plot.

### Pooling HR in cohort studies

**Fig. 7:**
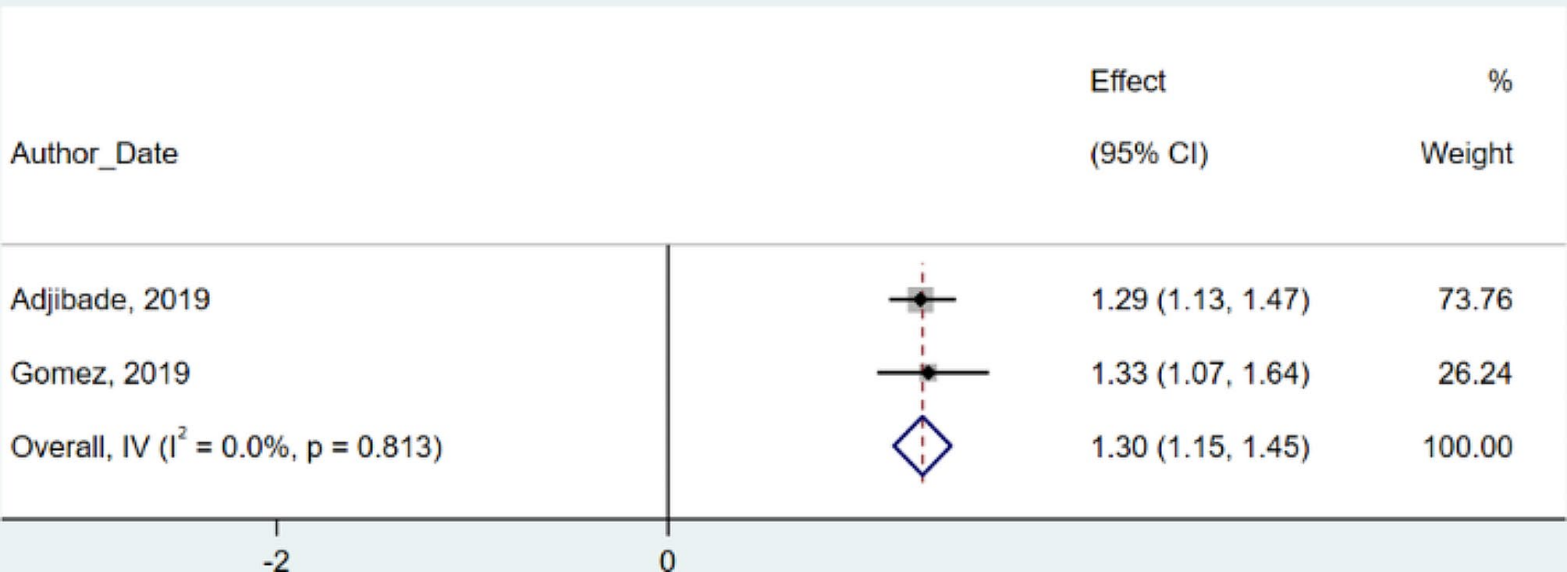
Junk food consumption and risk of depression in cohort studies

Aggregating two cohort studies with 41,637 participants showed an HR of 1.30 (95% CI: 1.15 to 1.45) for depression, demonstrating a significant risk increase (Fig. 7). Remarkably, these studies showed no heterogeneity (I² = 0.0%, *p* = 0.81) or publication bias.

## Discussion

The meta-analysis reported in the present study showed that high consumption of junk foods was significantly associated with increased risks of depression. In addition, higher junk food consumption was associated with increased odds of depression and psychological stress. This association between consumption of food with low nutritional value and mental health was demonstrated in multiple studies on different populations and cultures [17–19].

Meta-analysis of prospective studies showed that increased risk of subsequent depression and adverse mental health outcomes were correlated with higher ultra-processed food intake [20]. According to meta analysis incorporating seven studies, junk food consumption increased the risk of experiencing mental illness symptoms [21]. For example, one study reporting outcomes for 1591 adults, demonstrated that high consumption of fast foods and processed foods was associated with anxiety, nervousness, restlessness, lack of motivation and depressive symptoms [22]. In another study, weight gain due to unhealthy eating was associated with deterioration in mental health in 404 adults during the second year of the COVID-19 pandemic [23]. Our findings are consistent with a recent systematic review and dose-response meta-analysis that included 26 studies and 260,385 participants from twelve countries, which showed that ultra-processed food consumption increased risk of depression [24].

Epidemiological data suggests that unhealthy food consumption may be associated with poorer mental health through its adverse effects on inflammatory processes, nutritional status, and neurotransmitter function. Inflammation has previously been associated with underlying biological bases for depression [25]. Several observational and meta-analysis studies have demonstrated an inverse association between the consumption of healthy foods including vegetables, fruits, whole-grain and fish, with depressive symptoms [26–29]. Healthy dietary patterns include a significant amount of tryptophan, an essential amino acid and precursor to serotonin; evidence shows that reduction in the availability of serotonin is associated with depression [30, 31].

The adoption of western dietary patterns that regularly include junk foods and fast foods can increase the probability of developing inflammatory and cardiovascular diseases. Inflammatory conditions are related to mental health disorders including depression, stress and anxiety [32, 33]. In addition, life stressors may augment the interconnection between depressive mood and unhealthy dietary patterns through activation of the brain’s reward system by foods that are high in sugar, fat, and salt [34].

There is also evidence that brain-derived neurotrophic factor (BDNF) may be reduced by consumption of a high fat diet. BDNF is associated with supporting existing neurons and the production of new neurons and implicated in the pathogenesis of depressive disorder. A reduction in BDNF impairs synaptic and cognitive function and neuronal growth, contributing to the development of psychological disorders [35]. Western-type diets include a higher amount of polyunsaturated omega-6 fatty acids, which increase proinflammatory eicosanoids, and decrease BDNF and neuronal membrane fluidity [36]. This suggests that the adverse effects of junk and fast foods on mental health might be associated with the high content of unhealthy fats contained in these foods [4]. Moreover, intake of high amounts of sugar through consumption of sweet drinks and snacks can lead to endothelial dysfunction, inflammation, and exaggerated insulin production that may also influence mood [37–40].

Mood disorder may itself influence diet, with some studies reporting that patients with depression consume a large amount of carbohydrate-fat-rich foods during their depressive episodes [41–43]. Serotonin, an important neurotransmitter for regulating mood, may play a prominent role in this respect given that the sole source of its precursor, tryptophan, is through the diet [44].

The consumption of ultra-processed foods is positively correlated with unhealthy eating habits, including lower intake of fruits and vegetables and higher intake of sweet foods or beverages [8, 45]. It is notable that ultra-processed foods contain additives as well as molecules that are generated by high-temperature heating. These can alter gut microbiota composition and reduce nutrient absorption [46]. Some studies have explored the association between the gut microbiome and mental health [47–49], with animal studies suggesting that food additives might increase symptoms of and susceptibility to anxiety and depression via changes of gut microbiota composition [50, 51].

The present paper found that the outcomes of studies selected for the meta-analysis were not always in agreement. This may have been due to confounding factors such as past history of depression or negative life events not being included in the analysis, differences in study designs, sample sizes or population characteristics, non-homogeneous assessment of dietary patterns, and inconsistencies in the evaluation of psychological disorders including the use of different diagnostic criteria to define mental health status.

On the other side, some studies have reported that mental health disorders including depression and psychological stress may reduce an individual’s motivation to eat healthy foods and sometime lead to overeating [17], skipping main meals and replacing them with high calories foods [30]. Some individuals consume high energy and fatty foods during stressful situations, choosing these more palatable foods as an unconscious or deliberate strategy to change their energy levels and mood [52, 53]. Stress affects neuroendocrine function by activating the hypothalamic-pituitary-adrenal (HPA) axis, increasing the secretion of glucocorticoids. These change glucose metabolism, promote insulin resistance, and alter the secretion of appetite-related hormones. All of these factors contribute to the propensity to eat more high-calorie palatable food [12]. However, there are also studies that report no differences in eating patterns under stressful and non-stressful conditions [54, 55]. The analysis presented in the present study cannot be used to demonstrate causality. On the basis of the evidence, it is plausible that there is a bidirectional relationship between junk food consumption and mental health [17]. It remains unclear whether the quality of food choices affects susceptibility to poorer mental health outcomes, and/or the experience of unpleasant emotions influences the quality of food selection [30]. Evidence for a causal pathway is unclear and needs to be further investigated in well-controlled longitudinal studies. Our meta-analysis on cross-sectional studies showed an association between junk food consumption and increased odds of having stress and depression. Besides, meta-analysis on cohort studies demonstrated that junk foods consumption increases the risk of developing stress and depression.

### Strengths and limitations

As the main strength of our study, we have comprehensively and specifically evaluated earlier findings regarding the association between junk food consumption and mental health status in adults. The present study has some limitations arising from the studies selected for meta-analysis. Inconsistencies in design of studies such as the ways that diet is assessed using different dietary questionnaire tools, the influence of seasonal and hormonal variations of depressive symptoms, and the use of different diagnostic criteria for defining mental health status is one of the limitations of this study. Despite the association shown between consumption of junk foods and mental health disorders, the strength of the associations and number of documents included in this study is unable to demonstrate causality.

## Conclusion

The present study supports the conclusion that consumption of junk foods that are high in fat and sugar content and of low nutritive value are associated with poorer mental health in adults. Further studies utilizing a longitudinal design are needed to better determine the directionality and effect size of junk food consumption on psychological disorders. Moreover, more studies are warranted to assess the mechanisms involved in this relationship to provide scientific support for changes in public health policies.

## Data Availability

Data will be made available on request from the authors.
